# Transforming healthcare through regenerative medicine

**DOI:** 10.1186/s12916-016-0669-4

**Published:** 2016-08-10

**Authors:** Zita M. Jessop, Ayesha Al-Sabah, Wendy R. Francis, Iain S. Whitaker

**Affiliations:** 1 Reconstructive Surgery & Regenerative Medicine Group, Swansea University, Swansea, UK; 2 The Welsh Centre for Burns & Plastic Surgery, Morriston Hospital, Swansea, UK; 3 Institute of Life Sciences, Swansea University Medical School, Swansea University, Swansea, UK

**Keywords:** Regenerative medicine, Cell therapy, Tissue engineering, Stem cells, Clinical translation

## Abstract

Regenerative medicine therapies, underpinned by the core principles of rejuvenation, regeneration and replacement, are shifting the paradigm in healthcare from symptomatic treatment in the 20th century to curative treatment in the 21st century. By addressing the reasons behind the rapid expansion of regenerative medicine research and presenting an overview of current clinical trials, we explore the potential of regenerative medicine to reshape modern healthcare.

## Background

The current dilemmas for modern day healthcare, such as an aging population and the increasing prevalence of chronic diseases, require solutions that limit organ dysfunction and tissue degeneration and which potentially offer replacement. This was first addressed through transplantation, a field that advanced rapidly in the 1950s through a combination of surgical innovations and fundamental scientific breakthroughs in immunosuppression [1]. In contrast to the allogenic replacement of transplantation, regenerative medicine seeks to apply stem cell research with developmental biology principles to regenerate cells, tissues and organs de novo [2].

**Fig. 1:**
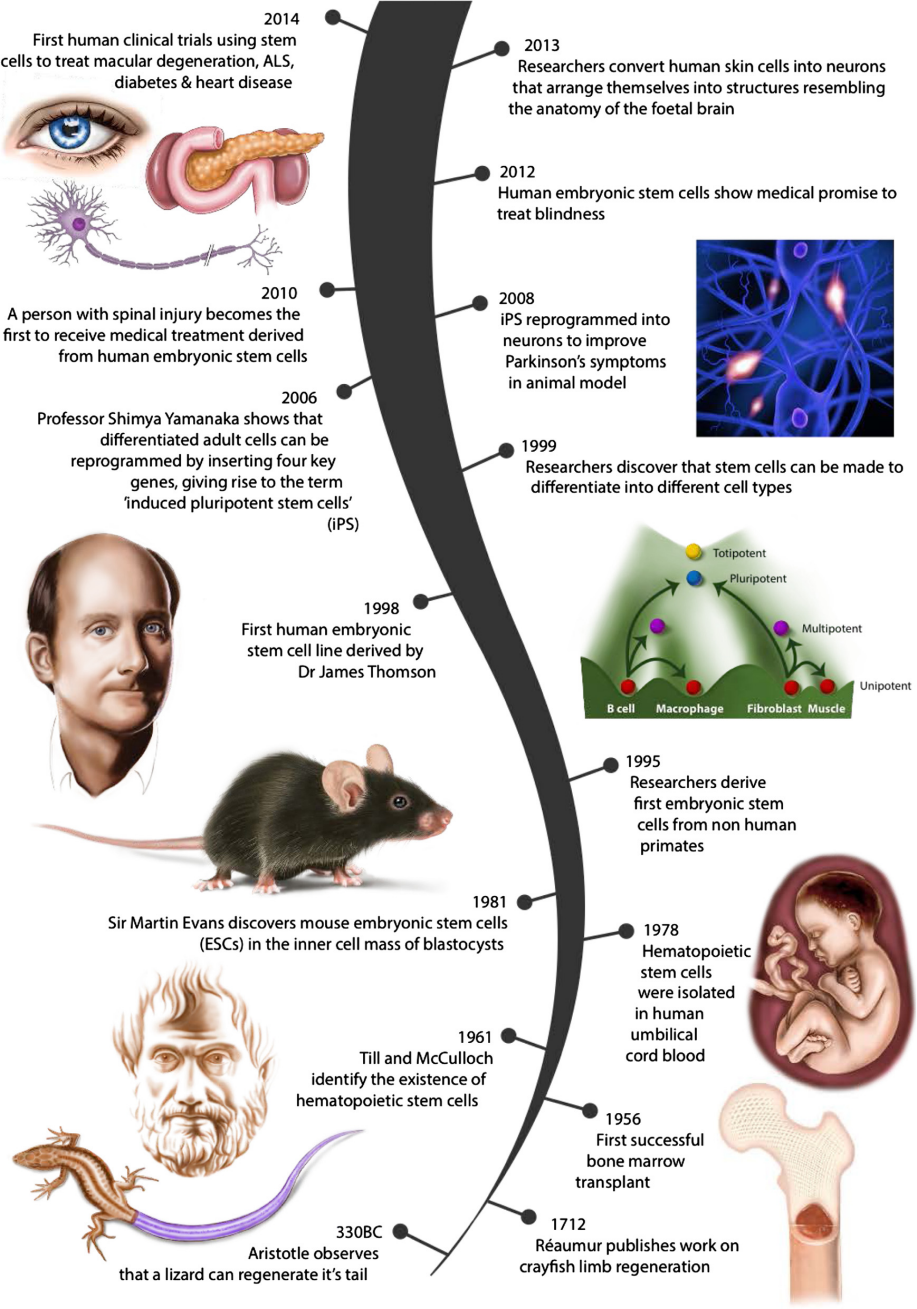
Regenerative medicine origins

The regenerative medicine research field resulted from the convergence of multiple scientific avenues, such as successful culture of cells in the laboratory [3], identification, characterization and differentiation of stem cells [4–7], and an improved understanding of developmental and molecular biology [8], to conceivably allow control of the intracellular and extracellular environment to promote tissue and organ formation in the laboratory (Fig. 1).

Regenerative medicine has been recognized worldwide as a developing research field that offers the potential to revolutionize patient care in the 21^st^ century [9]. The prospect of addressing massive healthcare markets, such as cardiovascular disease, neurological conditions or chronic metabolic diseases (e.g. end-stage renal disease or diabetes), means that there has been sustained scientific, public and commercial interest despite early setbacks and slow progress.

## Expansion and potential impact of regenerative medicine

Demand for regenerative medicine products has been driven by an increase in degenerative and chronic diseases which place cost pressures on healthcare providers, combined with advances in new technologies such as nanotechnology, bioengineering and stem cell therapy [10]. Long-term cell, tissue and organ replacement will not only provide an alternative to transplantation [11], but will also provide therapeutic options for degenerative conditions (e.g. neurodegenerative conditions (Parkinson’s), stroke and heart failure), which are currently only managed through palliation [12, 13].

According to the World Regenerative Medicines Market forecast for 2013–2020 [14], the global regenerative medicines market for small molecules and biologics, gene therapy and cell therapy is expected to reach $67.5 billion by 2020, which is an increase of $51.1 billion from 2013, thus reflecting its commercial potential. Governments across Europe and the US, as well as their medical research councils, have identified tissue engineering and regenerative medicine at the top of their research priorities [9]. Removal of previous restrictions in embryonic stem cell research in 2009 by the Obama organization is predicted to contribute to further considerable growth within the field as well as improved potential for clinical translation [15].

## Clinical trials in regenerative medicine

The expansion of regenerative medicine as a scientific discipline, with its core principles of rejuvenation, regeneration and replacement (the 3R’s), is shifting the paradigm in healthcare from symptomatic treatment in the 20th century to curative treatment in the 21st century [13]. This is evidenced by the rapid increase in regenerative medicine clinical trials in each specialty [16, 17], which can be broadly classified as using either cell- or tissue-based products (Table 1). The Food and Drug Administration in the US and the European Medicines Agency have more complex classification systems of regenerative medicine products, including cellular therapy, gene therapy, stimulators of endogenous repair, biologic-device combination products, and human tissue and xenotransplantation [18]. Broadly, the regulatory requirements can be based on the pillars of sterility, stability and potency, and these need to be addressed prior to successful clinical translation in the future (Table 2).

**Table 1:** Applications of regenerative medicine therapies in different medical specialties

Medical Speciality	Pathology	Cell/Tissue Therapy	Clinical Trial Phase	Patient numbers	Clinical Trial Study
			Pubmed Clinical Trial database		
Neurology	Parkinson’s	Fetal porcine cells Transplantation of embryonic dopamine neurons	I, II	34	Fink 2001; Freed 2001
	Paraplegia, Spinal cord injuries	MSCs transplanted directly into injured spinal cord. Bone marrow nucleated cells injected intrathecally and intravenously coupled with MSC infusion by lumbar puncture	I, II	80	Park 2012; Jarocha 2015
	Multiple Sclerosis	IV infusion of MSCs Haemopoietic stem cell transplants	I, II	30	Connick 2012
Cardiology	Ischaemic cardiomyopathy, heart failure	Transendocardial injection of MSC derived from BM or adipose tissue Intracoronary injection of cardiac stem cells IV infusion of MSC	I, II	104	Heldman 2014; Hare 2014; Chugh 2012; Perin 2014
Respiratory	Idiopathic pulmonary fibrosis	IV infusion of placental- Chambers derived MSC	I	8	Chambers 2014
	Chronic lung disease	IV infusion of HLA-matched allogeneic MSCs derived from BM/umbilical cord	I,II	62	Weiss 2013
Rheumatology	Osteoarthritis	Intra-articular injection of autologous or allogeneic MSC	I, II, III	104	Orozco 2013; Jo 2014
	Osteogenesis Imperfecta	Allogeneic bone marrow derived MSC Haemopoietic stem cell transplant plus MSC infusion	I	8	Horwitz 2002; Horwitz 2002
Orthopaedics	Fracture healing; Joint resurfacing; Osteoporosis	MSC combined with/without calcium sulphate Allogenic bone graft containing stem cells G-CSF-mobilised Haemopoietic stem cells with collagen scaffold for non-union fracture healing	I,II	96	Kuoroda 2014; Jones 2015; Bajada 2007
Haematology	Hematopoietic stem cell transplant (HSCT); Graft versus Host Disease (GvHD)	Prochymal (MSC) for severe refractory acute GvHD MSC infused with or following hematopoietic stem cell transplant	I, II, III	240	Prasad 2011; Ringden 2006; Perez-Simon 2011
Opthalmology	Macular degeneration	ESC-derived retinal pigment epithelium	I	2	Schwartz 2012
Gastroenterology	Liver cirrhosis; Decompensated liver disease	MSC injected into peripheral or portal vein Autologous bone marrow mononuclear cells infused IV for liver cirrhosis UC-MSC IV in fusion in decompensated liver disease	I,II	45	Kharaziha 2009; terai 2006; Zhang 2012
	Crohn’s disease	Autologous hematopoietic)stem cell transplantation for refractory Crohn’s	I, II, III	98	Oyama 2005
Endocrinology	Diabetes (type I & 2)	Stem cell educator therapy with cord blood derived stem cells for insulin resistant type II diabetes Hematopoietic stem cell transplantation for new onset type I diabetes	I, II	65	Zhao 2013, D’Addio 2014
Nephrology/Urology	Kidney transplant rejection	MSC based therapy to prevent rejection in living-related kidney transplants	I, II	159	Tan 2012

*MSC* mesenchymal stem cells, *BM* Bone marrow, *ESC* Embryonic Stem cells, *iPSC* induced Pluripotent stem cells, *IV* intravenous, *G-CSF* granulocyte-colony stimulating factor, *3D* 3-dimensional, *UC* umbilical cord

**Table 2:** Overview of testing of regenerative medicine products to validate sterility, stability and potency

Pillar	Obstacles	Method	Reference
Sterility	Sterility testing	Direct inoculation test in aerobic and anaerobic media	[32]
Stability	Chromosomal stability	Karyotyping	[33]
	Cell metabolism	Mitochondrial bioenergetics	[34]
	Safety	Animal testing to investigate interactions between native tissue and product	[35]
Potency	Cell identity	Flow cytometry and immunohistochemical analysis	[36]
	Reproducibility	Purity and viability of cell population	[37]
	Cell tracking	Fluorescent/superparamagnetic iron oxide cell labeling prior to animal implantation	[35]

Cell-based therapies work either via stimulation of endogenous repair through extracellular factors or differentiation and functional replacement of endogenous cell types [17]; they include stem cell implantation or infusion to treat hematopoietic diseases, cardiac conditions and Parkinson’s disease. Most of the pioneering work has been performed using haematopoietic stem cells due to the early bone marrow transplant work, making them the most well-studied stem cell type [19]. In particular, adult mesenchymal stem cells have gained interest as they avoid the ethical concerns of using embryonic stem cells, can be rapidly expanded in vitro and avoid immunogenicity. Studies have shown contradictory results on the efficacy of the transplanted cells, with patient variability with regards to response (Table 1); further work is needed to elucidate cell identity and health to ensure patient safety (Table 2).

The tissue engineering strand of regenerative medicine incorporates cells with biodegradable scaffolds to engineer replacement tissues like dermis or cartilage [20] and whole organs such as trachea and bladder [21, 22]. Limitations of synthetic polymer scaffolds, such as infection, extrusion and degradation product toxicity, have encouraged interest in decellularised matrices as well biologics for use as scaffolds as one of the more effective ways of replicating native tissue anisotropy [21, 22]. Decellularised matrices provide durability, enhanced integration and biocompatibility whilst avoiding allosensitization [21]. This may explain why many of the significant breakthroughs and first-in-man studies have utilized this technique combined with autologous cell-seeding with some success [21–23], and even showed promise in vitro for more complex structures such as pulmonary and aortic valves as well as whole organs such as heart and liver [24, 25]. However, despite early interest and investment in tissue engineering research, with annual R&D spending estimated at US$580 million, initial clinically applicable product release has been slow but steady [26].

## Controversies in the field

The regenerative medicine field has been shrouded in controversy. Significant potential gains have led to several high profile allegations of research misconduct [27, 28]. There is also a growing stem cell tourism industry based on unproven treatments that aims to capitalize on stem cell hype [29, 30]. Desperate patients would rather approach private clinics offering experimental stem cell treatments, with unproven safety and efficacy profiles, than wait for outcomes of clinical trials [30]. Media coverage and direct advertising of stem cell therapies as well as the political, ethical and religious controversies surrounding human embryonic stem cells, can contribute not only to increased public awareness but also inflated expectations of regenerative medicine products, and there continues to be a significant gap between the perceived and realistic benefits [31]. A concerted effort from the scientific community as well as robust outcome data from clinical trials will be needed to temper unrealistic claims [16, 17].

## Conclusion

Medical breakthroughs often require the convergence of multiple scientific advances for which interdisciplinary collaboration is fundamental. Similar to transplant medicine, regenerative medicine requires the convergence of a number of scientific disciplines, including stem cell biology, developmental and molecular biology, engineering and biomaterials. Despite media hype, scientific overclaim and unrealistic expectations, which have been previously witnessed for a number of healthcare technologies, regenerative medicine continues to make steady progress reflected by the increasing number of clinical trials [16, 17]. Significant potential has been demonstrated in the cell therapy field to treat haematological, neurological and rheumatological conditions. The tissue engineering field, although holding great promise, still has some way to develop before the excitement surrounding novel biofabrication strategies, such as 3D bioprinting, is translated to patient care. The fast moving and versatile field of regenerative medicine is at the cutting edge of translational research and could shift the paradigm in healthcare from symptomatic to curative treatment. *BMC Medicine* is very interested in breakthroughs in regenerative medicine/stem cell therapy and submission of such relevant articles is encouraged.
